# Early Nutritional–Immune Biomarker Trajectories Following Gastrectomy for Gastric Cancer: A Prospective Longitudinal Cohort Study

**DOI:** 10.3390/nu18172862

**Published:** 2026-09-02

**Authors:** Catalin Dumitru Cosma, Vlad Olimpiu Butiurca, Dragos Molnar, Cosmin Nicolescu, Calin Molnar, Marian Botoncea

**Affiliations:** 1 Faculty of Medicine, George Emil Palade University of Medicine, Pharmacy, Sciences and Technology of Târgu Mureș, 540139 Târgu-Mureș, Romania; catalin.cosma@umfst.ro (C.D.C.); vladbutiurca@yahoo.com (V.O.B.); cosmin.nicolescu@umfst.ro (C.N.); molnar.calin@yahoo.com (C.M.); marian.botoncea@umfst.ro (M.B.); 2 General Surgery Clinic No.1, County Emergency Clinical Hospital of Târgu-Mureș, 540136 Târgu-Mureș, Romania

**Keywords:** gastric cancer, gastrectomy, nutritional recovery, longitudinal study, nutritional status, CONUT, postoperative nutrition, prospective cohort, enhanced recovery after surgery, malnutrition

## Abstract

Background: Nutritional deterioration is a common consequence of gastrectomy for gastric cancer and may persist despite standardized perioperative care. However, prospective longitudinal evidence describing the early course of postoperative nutritional recovery remains limited. This study aimed to characterize nutritional recovery trajectories following curative gastrectomy and to evaluate the influence of the extent of gastric resection on postoperative recovery. Methods: A prospective cohort of 239 consecutive patients undergoing curative-intent subtotal or total gastrectomy for gastric adenocarcinoma was followed between January 2022 and December 2025. The longitudinal complete-case analysis included 217 patients with available assessments at all three predefined time points: preoperatively (T0), at hospital discharge (T1), and three months after surgery (T3). Nutritional–immune biomarker trajectories were assessed preoperatively (T0), at hospital discharge (T1), and three months after surgery (T3) using serum albumin, total cholesterol, absolute lymphocyte count, and the Controlling Nutritional Status (CONUT) score. Longitudinal changes were evaluated using linear mixed-effects models with patient-specific random intercepts. Results: All evaluated nutritional–immune biomarkers changed significantly in an adverse direction after surgery, reaching their most unfavorable values at hospital discharge (all *p* < 0.001), followed by partial biochemical recovery at three months. Because albumin, lymphocyte count, and CONUT are influenced by the acute inflammatory and metabolic response to surgery, the discharge changes should not be interpreted as direct evidence of acute malnutrition. Nevertheless, none of the evaluated biomarkers returned to its preoperative value. Recovery between discharge and three months represented 65.6% of the initial decline for serum albumin, 50.5% for absolute lymphocyte count, 51.7% for total cholesterol, and 60.9% for the CONUT score. After adjustment for age, sex, neoadjuvant chemotherapy, baseline body mass index, ASA status, pathological T3–T4 stage, and major postoperative morbidity, total gastrectomy was associated with a greater early decline in absolute lymphocyte count at hospital discharge (β = −165.7 cells/mm^3^; 95% CI, −204.9 to −126.6; *p* < 0.001). This difference was no longer present at three months (*p* = 0.955). No significant time-by-gastrectomy interactions were observed for albumin, cholesterol, or corrected CONUT. Conclusions: Nutritional recovery following gastrectomy is a dynamic and prolonged process characterized by marked early nutritional–inflammatory biomarker changes and incomplete biochemical restoration at three months. Resection extent was not independently associated with persistently less favorable albumin, cholesterol, or corrected CONUT trajectories. The findings describe early biochemical changes and should not be extrapolated to body composition, functional recovery, micronutrient status, quality of life, or long-term survivorship. These findings apply primarily to patients who survived and completed the three-month follow-up.

## 1. Introduction

Gastric cancer is still a major global health problem, with more than one million new cases and approximately 660,000 deaths each year. Despite substantial progress in prevention, diagnosis, and treatment, gastric cancer remains one of the leading causes of cancer-related mortality [1]. Although the incidence has slowly decreased in most parts of the world, the disease still contributes significantly to the burden of the healthcare system, especially in East Asia, Eastern Europe, and some areas of South America [2]. Radical gastrectomy with adequate lymphadenectomy according to the internationally accepted surgical standards proposed by the Japanese Gastric Cancer Association, European Society for Medical Oncology (ESMO), and National Comprehensive Cancer Network (NCCN) is the mainstay for curative treatment [3,4,5]. Better postoperative survival has been achieved by the improved surgical techniques, perioperative care, and multimodal oncologic treatment which has shifted the clinical attention from short-term morbidity to long-term functional and nutritional recovery [6]. 

Nutritional impairment is one of the most common and clinically relevant sequelae of gastric cancer and its surgical treatment. Due to decreased oral intake, metabolic changes associated with cancer, and systemic inflammation, a large proportion of patients are already in different stages of malnutrition before surgery. These abnormalities are frequently worsened after gastrectomy because of loss of gastric reservoir function, impaired mechanical digestion, accelerated gastrointestinal transit, altered hormonal regulation, and decreased absorption of essential nutrients [6,7,8]. Consequently, postoperative nutrition care has been integrated into enhanced recovery pathways, and the current ERAS recommendations emphasize early oral intake, structured nutritional assessment, and individualized nutritional support during the perioperative period [9]. The implementation of these strategies in the clinical setting has been correlated with better postoperative recovery and less treatment-related morbidity in specialized gastric cancer centers [10]. 

Growing evidence suggests that nutritional status is much more than a marker of caloric intake, as it is a reflection of the complex interaction between the metabolic reserve, systemic inflammation, and host immune competence. For this reason, some validated nutritional indices, such as the Prognostic Nutritional Index (PNI), Controlling Nutritional Status (CONUT) score, and Geriatric Nutritional Risk Index (GNRI), demonstrated significant prognostic value in patients who underwent gastrectomy [11,12,13]. Similarly, the major determinants of impaired functional recovery, reduced quality of life, and worse long-term survival, particularly after total gastrectomy, have been identified as postoperative vitamin B12 deficiency, chronic anemia, persistent body weight loss, and progressive sarcopenia [14,15,16,17]. These findings are in line with the concept that the postoperative nutritional rehabilitation is a long biological process far beyond the immediate postoperative phase.

The clinical relevance of postoperative nutritional impairment extends beyond changes in body weight or routine laboratory values. Persistent inadequate intake and altered gastrointestinal physiology may contribute to skeletal-muscle depletion and sarcopenia, which are associated with reduced physical performance, loss of functional independence, higher postoperative morbidity, impaired tolerance of systemic oncological treatment, and worse long-term outcomes. Gastrointestinal symptoms, including early satiety, dumping symptoms, reflux, nausea, diarrhea, and altered bowel function, may further restrict oral intake and negatively affect health-related quality of life. Consequently, nutritional recovery after gastrectomy should be regarded as a multidimensional process encompassing dietary intake, body composition, physical function, treatment tolerance, gastrointestinal symptom burden, and patient-reported well-being [15,16,17].

Prospective observational studies on postoperative recovery should be conducted and reported in accordance with established methodological standards to ensure transparency and reproducibility [18]. Nutritional recovery is a dynamic process, so longitudinal analyses require statistical methods that can accommodate repeated measurements and incomplete follow-up, while minimizing bias due to missing observations [19,20,21]. Also, proper sample size determination still remains important to have enough statistical power to detect clinically meaningful longitudinal changes [22]. 

Recent studies have shown that patients do not recover nutritionally in a uniform manner following surgery. Patients appear to follow different recovery trajectories, with variable patterns of restoration of body weight, body composition, and metabolic adaptation during postoperative follow-up [23]. Recent advances in perioperative nutritional therapy have demonstrated that oral nutritional supplementation is capable of significantly reducing postoperative weight loss after gastrectomy. These results have been confirmed by several systematic reviews and meta-analyses [24]. At the same time, the increased awareness of micronutrient deficiency after surgery has underlined the need for systematic monitoring of vitamin B12 status and timely supplementation after gastric resection [25,26]. Moreover, the preservation of skeletal muscle mass has become a relevant therapeutic goal, as postoperative sarcopenia has been proven to be strongly associated with functional decline and long-term oncological outcome [27]. Furthermore, perioperative immunonutrition has demonstrated promising effects in selected patient populations by reducing postoperative immune dysfunction and enhancing nutritional recovery [28,29]. Preoperative chronic systemic inflammation has also been associated with an increased risk of postoperative complications regardless of body composition, highlighting the close interaction between nutritional and inflammatory pathways [30]. Contemporary nutritional management thus goes beyond caloric replacement to include sarcopenia prevention, micronutrient deficiencies, and persistent metabolic dysfunction throughout survivorship [31,32,33].

In addition to nutritional assessment, there is increasing interest in systemic inflammatory biomarkers as potential markers of postoperative recovery. Composite indexes such as the systemic immune–inflammation index (SII) and inflammatory ratios based on routine blood tests, such as neutrophil-to-lymphocyte ratio, platelet-to-lymphocyte ratio, and lymphocyte-to-monocyte ratio, have been consistently found to be of prognostic significance in gastric cancer [11,34,35,36,37,38]. Postoperative changes in body composition, especially skeletal muscle depletion and de novo sarcopenia, have been associated with poor long-term survival, underlining the importance of combined assessment of nutritional and functional recovery rather than body weight evaluation alone [39,40]. Nutritional status, dietary intake, gastrointestinal function, and gastric emptying continue to change for many months after surgery, with longitudinal studies further supporting the concept of recovery as a dynamic process rather than a single postoperative endpoint [41,42]. Current ESPEN recommendations and later systematic reviews support prolonged nutritional monitoring, tailored enteral supplements, and organized nutritional support in the post-discharge period to optimize recovery after upper gastrointestinal surgery [43,44,45]. Randomized clinical trials have demonstrated that oral nutritional supplementation after surgery decreases body weight loss after gastrectomy and may have lasting long-term benefits beyond the early postoperative period [46,47,48].

Although long-term nutritional impairment, body-composition changes, sarcopenia, micronutrient deficiencies, and quality-of-life consequences after gastrectomy are well documented, fewer studies have examined the short-term evolution of routinely available nutritional–immune biomarkers using repeated measurements and longitudinal models that account for differences in tumor burden and postoperative morbidity. Such biomarkers do not provide a comprehensive assessment of nutritional or functional recovery but remain readily accessible in routine clinical practice and may help characterize early postoperative biological adaptation.

Accordingly, the primary objective of the present prospective longitudinal cohort study was to characterize early trajectories of serum albumin, absolute lymphocyte count, total cholesterol, and the CONUT score at three predefined perioperative assessment points. A secondary objective was to determine whether these trajectories differed between subtotal and total gastrectomy after adjustment for age, sex, baseline body mass index, ASA status, neoadjuvant chemotherapy, pathological tumor stage, and postoperative morbidity. It was hypothesized that apparent differences according to resection extent might be influenced partly by baseline case mix and postoperative complications. The study was not designed as a comprehensive evaluation of body composition, physical function, micronutrient status, quality of life, or long-term survivorship.

## 2. Materials and Methods

### 2.1. Study Design and Patient Population

This prospective longitudinal observational cohort study was conducted at the General Surgery Clinic No. 1, County Emergency Clinical Hospital of Târgu Mureș, Romania, in collaboration with the George Emil Palade University of Medicine, Pharmacy, Science, and Technology of Târgu Mureș. Consecutive adult patients undergoing curative-intent gastrectomy for histologically confirmed gastric adenocarcinoma between January 2022 and December 2025 were prospectively enrolled and followed according to a predefined institutional protocol.

The primary objective was to characterize the early longitudinal trajectories of routinely available nutritional–immune biomarkers following gastrectomy using serial measurements obtained at predefined perioperative assessment points. The secondary objective was to evaluate whether these trajectories differed between subtotal and total gastrectomy after adjustment for clinically relevant differences in baseline case mix, pathological tumor stage, and postoperative morbidity. These biochemical trajectories were considered components of early postoperative biological recovery and not a comprehensive assessment of nutritional status, functional recovery, or long-term survivorship.

Patients were eligible if they were aged ≥18 years and underwent subtotal or total gastrectomy with curative intent for histologically confirmed gastric adenocarcinoma. Patients undergoing palliative procedures, emergency surgery, surgery for recurrent gastric cancer, or multivisceral resections performed for non-curative purposes were excluded. Completion of all scheduled postoperative assessments was not an eligibility criterion for the full prospective cohort.

Pre-existing malnutrition or an abnormal preoperative CONUT score was not an exclusion criterion because the study aimed to evaluate postoperative trajectories across the spectrum of baseline nutritional risk. No patient was excluded solely because of a low albumin concentration, lymphopenia, hypocholesterolemia, low body mass index, or elevated CONUT score. Baseline nutritional–immune biomarker values were retained in the analysis and constituted the reference measurements for the longitudinal models. No additional exclusion criteria were applied solely on the basis of comorbid conditions potentially affecting the evaluated biomarkers. These factors were documented when available and were considered when interpreting the nonspecific nutritional–inflammatory nature of the laboratory outcomes.

A total of 239 consecutive eligible patients constituted the full prospective cohort, including 160 patients undergoing subtotal gastrectomy and 79 undergoing total gastrectomy. Complete assessments at T0, T1, and T3 were available for 217 patients, who constituted the complete-case cohort used for the longitudinal biomarker analysis.

The study was designed, conducted, and reported according to the Strengthening the Reporting of Observational Studies in Epidemiology (STROBE) recommendations for prospective observational studies.

### 2.2. Surgical Treatment and Perioperative Nutritional Management

The extent of gastric resection (subtotal or total gastrectomy), lymphadenectomy, and reconstructive procedure were determined according to current international gastric cancer treatment guidelines, tumor characteristics, and intraoperative findings, following multidisciplinary evaluation. All operations were performed by experienced upper gastrointestinal surgeons using standardized institutional techniques.

Perioperative management followed a standardized institutional Enhanced Recovery After Surgery protocol. The protocol included preoperative nutritional assessment, avoidance of prolonged fasting, early postoperative mobilization, multimodal analgesia, and early oral feeding whenever clinically appropriate. Nutritional counselling was provided during hospitalization, and patients received standardized postoperative dietary recommendations before discharge. Oral nutritional supplements were prescribed according to nutritional risk, oral intake, clinical tolerance, and the institutional nutritional protocol.

Standardization applied to the inpatient pathway and discharge recommendations. The amount and duration of oral nutritional supplementation consumed after discharge, adherence to dietary recommendations, attendance at outpatient dietetic counselling, quantitative dietary intake, and use of pancreatic enzyme replacement therapy were not systematically documented during the three-month follow-up. These exposures could therefore not be incorporated as time-varying covariates in the longitudinal models.

Because perioperative care was standardized for all participants, observed differences in nutritional recovery predominantly reflected patient-related biological variability rather than differences in postoperative management.

### 2.3. Nutritional Assessment and Longitudinal Follow-Up

Nutritional recovery was evaluated prospectively using serial anthropometric and laboratory assessments performed at three predefined time points:

- T0: preoperative evaluation (within 48 h before surgery).
- T1: the morning of hospital discharge, corresponding to postoperative day 7 [5,6,7,8,9,10,11,12,13,14,15]. Blood sampling was performed before discharge according to the institutional laboratory protocol.
- T3: postoperative outpatient follow-up at 3 months.

At each assessment, fasting venous blood samples were collected according to institutional laboratory protocols. The principal biochemical variables included serum albumin concentration, total cholesterol concentration, and absolute peripheral lymphocyte count. These variables were evaluated as routinely available nutritional–immune biomarkers rather than specific diagnostic measures of malnutrition. In the acute postoperative setting, serum albumin is a negative acute-phase protein and may be influenced by systemic inflammation, capillary permeability, perioperative fluid shifts, hepatic protein-synthesis reprioritization, and illness severity. Absolute lymphocyte count and total cholesterol may likewise be affected by surgical stress, inflammation, metabolic changes, and nutritional intake. Accordingly, their longitudinal changes were interpreted as reflecting the combined nutritional, inflammatory, immune, and metabolic response to gastrectomy.

Baseline body mass index (BMI) was calculated as weight (kg)/height^2^ (m^2^) using standardized preoperative measurements. Percentage postoperative body-weight loss was recorded during follow-up and used as an additional indicator of nutritional deterioration.

As a secondary composite assessment, the CONUT score was calculated from serum albumin, absolute lymphocyte count, and total cholesterol according to the original scoring system. Because each component may be influenced by systemic inflammation and surgical stress, particularly during the early postoperative period, CONUT was interpreted as an inflammation-sensitive nutritional–immune composite index rather than as a specific measure of acute malnutrition.

Clinical variables collected prospectively included demographic characteristics, comorbidities, American Society of Anesthesiologists (ASA) physical status classification, neoadjuvant chemotherapy, operative details, pathological staging, postoperative complications, and duration of hospital stay.

### 2.4. Study Outcomes

The primary outcome was the longitudinal trajectory of routinely available nutritional–immune biomarkers following gastrectomy, assessed through temporal changes in serum albumin, total cholesterol, and absolute lymphocyte count between the predefined assessment points.

Secondary outcomes included postoperative body-weight loss, longitudinal evolution of the CONUT score, postoperative complications classified according to the Clavien–Dindo classification, duration of hospitalization, and factors independently associated with less favorable biomarker recovery.

Recovery trajectories were analyzed at the population level while accounting for repeated within-patient measurements, allowing characterization of the temporal evolution of nutritional status rather than isolated postoperative observations.

### 2.5. Statistical Analysis

Continuous variables were expressed as mean ± standard deviation (SD) or median with interquartile range (IQR), according to data distribution assessed using the Shapiro–Wilk test. Categorical variables were summarized as absolute frequencies and percentages.

Longitudinal changes in serum albumin, absolute lymphocyte count, total cholesterol, and CONUT score were evaluated using linear mixed-effects models with patient-specific random intercepts. Time was treated as a categorical variable, with the preoperative assessment as the reference category. Gastrectomy type and separate gastrectomy-type-by-time interaction terms were included to estimate whether changes at hospital discharge and three months differed between subtotal and total gastrectomy.

The adjusted model included age, sex, neoadjuvant chemotherapy, baseline body mass index, ASA III–IV status, pathological T3–T4 stage, and major postoperative morbidity, defined as Clavien–Dindo grade III or higher. Because tumor stage and postoperative morbidity could affect postoperative biomarker changes, stage-by-time and major-complication-by-time interactions were included. A sensitivity analysis was performed by replacing major morbidity with the occurrence of any postoperative complication. Adjusted coefficients are presented with 95% confidence intervals and two-sided *p*-values.

To assess potential attrition bias, baseline characteristics were compared between patients included in the complete-case longitudinal analysis and those without complete three-month assessments. Continuous variables were compared using the independent-samples *t*-test or Mann–Whitney U test, according to their distribution, while categorical variables were compared using the chi-square test or Fisher’s exact test, as appropriate. This exploratory comparison included baseline body mass index, ASA III–IV status, and preoperative CONUT score.

CONUT scores were verified and recalculated directly from serum albumin, absolute lymphocyte count, and total cholesterol using the original validated scoring algorithm.

The longitudinal biomarker analysis was performed as a complete-case analysis including patients with available measurements at all three predefined time points. Of the 239 eligible patients, 22 did not have complete three-month follow-up because of 90-day mortality (*n* = 5) or non-attendance at the three-month assessment (*n* = 17). No imputation was performed because the unavailable three-month measurements resulted from death or loss to follow-up rather than sporadically missing laboratory values. The potential for informative attrition and survivorship bias was considered when interpreting the longitudinal estimates. Statistical significance was defined as a two-sided *p* value < 0.05. Statistical analyses were performed using EasyMedStat (EasyMedStat SAS 4.4, Paris, France).

The extent of gastric resection was determined by the multidisciplinary and operative assessment according to tumor location, longitudinal tumor extent, and the possibility of obtaining an oncologically adequate proximal margin. Subtotal gastrectomy was performed when complete oncological resection with an adequate proximal margin could be achieved while preserving a functional gastric remnant. Total gastrectomy was selected for proximal tumors, diffuse or extensive gastric involvement, or situations in which an adequate proximal margin and functional remnant could not otherwise be obtained. The procedure was not assigned for research purposes.

### 2.6. Ethical Considerations

The study was conducted in accordance with the ethical principles of the Declaration of Helsinki and received approval from the Institutional Ethics Committee of the County Emergency Clinical Hospital, Târgu Mureș (Decision No. 30104/7 October 2021). Written informed consent was obtained from all participants before study enrolment. Patient confidentiality was maintained throughout the study, and all data were anonymized before statistical analysis.

## 3. Results

### 3.1. Patient and Treatment Characteristics

**Figure 1:**
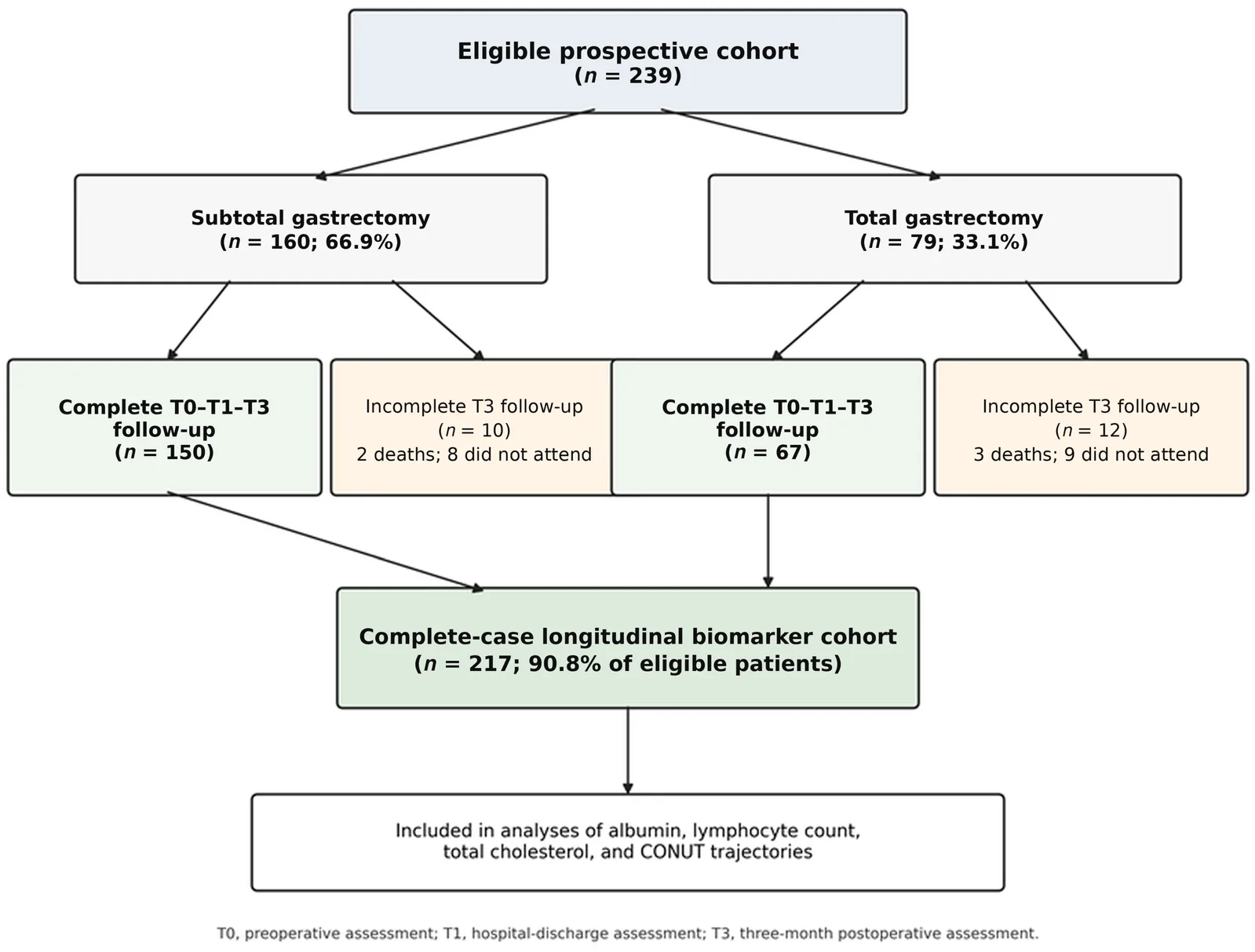
Study flow diagram. Of 239 eligible patients prospectively enrolled, 217 completed the predefined assessments at T0, T1, and T3 and were included in the complete-case longitudinal biomarker analysis. Five patients died within 90 days after surgery, and seventeen did not attend the three-month assessment.

Between January 2022 and December 2025, 239 eligible patients were prospectively enrolled, including 160 patients undergoing subtotal gastrectomy and 79 undergoing total gastrectomy. Complete assessments at T0, T1, and T3 were available for 217 patients: 150 after subtotal gastrectomy and 67 after total gastrectomy. Twenty-two patients did not contribute to the complete-case trajectory analysis. Five patients died within 90 days, including two after subtotal gastrectomy and three after total gastrectomy. Seventeen patients did not attend the three-month assessment, including eight after subtotal gastrectomy and nine after total gastrectomy. Follow-up completeness was therefore 90.8% overall, 93.8% after subtotal gastrectomy, and 84.8% after total gastrectomy. The mean age was 61.4 ± 10.6 years, 128 patients (59.0%) were male, and the mean preoperative body mass index was 23.8 ± 3.5 kg/m^2^. Neoadjuvant systemic treatment had been administered to 83 patients (38.2%) [Figure 1].

At the three-month assessment, complete data were available for 217 of the 239 enrolled patients. The 22 patients without complete three-month data comprised five deaths within 90 days, including two deaths during the index hospitalization, and 17 patients who did not attend follow-up. Compared with the 217 patients included in the complete-case analysis, these 22 patients did not differ significantly in baseline body mass index, ASA III–IV status, or preoperative CONUT score (all *p* > 0.05).

Patients undergoing total gastrectomy presented with a more advanced clinical and operative profile than those treated by subtotal gastrectomy. Neoadjuvant treatment was more frequent in the total gastrectomy group (53.7% vs. 31.3%; *p* = 0.002), as was pathological T3–T4 disease (74.6% vs. 48.7%; *p* < 0.001). Total gastrectomy was also associated with a longer operative time (244.7 ± 40.3 vs. 201.2 ± 23.8 min; *p* < 0.001) and a higher frequency of recorded postoperative complications (83.6% vs. 48.0%; *p* < 0.001) [Table 1].

Baseline demographic, clinical, oncological, and perioperative characteristics of the complete-case cohort are presented separately for subtotal and total gastrectomy. Because the extent of resection was determined by tumor location and oncological requirements rather than random allocation, the two procedure groups were not fully comparable. Patients undergoing total gastrectomy had a higher prevalence of pathological T3–T4 disease and neoadjuvant treatment and experienced a greater overall postoperative complication burden. These imbalances were considered in the adjusted longitudinal analyses.

### 3.2. Longitudinal Evolution of Nutritional–Immune Biomarkers

All three principal nutritional–immune biomarkers deteriorated significantly between the preoperative assessment and hospital discharge, followed by partial recovery at the three-month follow-up. Nevertheless, none had returned fully to baseline values by T3.

Mean serum albumin decreased from 3.83 ± 0.51 g/dL at T0 to 3.31 ± 0.59 g/dL at T1, subsequently increasing to 3.65 ± 0.56 g/dL at T3. The overall effect of time was statistically significant (*p* < 0.001). Bonferroni-adjusted pairwise comparisons confirmed significant differences between T0 and T1, T1 and T3, and T0 and T3, with all comparisons yielding *p* < 0.001.

Absolute lymphocyte count followed a similar pattern, declining from 1715 ± 614 cells/mm^3^ at T0 to 1369 ± 609 cells/mm^3^ at T1, before increasing to 1544 ± 619 cells/mm^3^ at T3. The longitudinal time effect was significant (*p* < 0.001), and each pairwise comparison remained significant after adjustment (all *p* < 0.001).

Mean total cholesterol decreased from 180.1 ± 39.3 mg/dL preoperatively to 154.4 ± 40.1 mg/dL at discharge, followed by an increase to 167.7 ± 40.0 mg/dL at three months. The overall temporal variation was significant (*p* < 0.001), with significant differences between all three assessment points (all adjusted *p* < 0.001).

The recalculated CONUT score increased from 1.99 ± 1.92 at T0 to 4.26 ± 2.51 at T1 and subsequently decreased to 2.88 ± 2.23 at T3. The overall effect of time and all adjusted pairwise comparisons were statistically significant (all *p* < 0.001). The early postoperative increase was interpreted as an adverse change in an inflammation-sensitive nutritional–immune composite rather than as direct evidence of acute malnutrition. Although the score improved by three months, it remained above its preoperative value.

**Figure 2:**
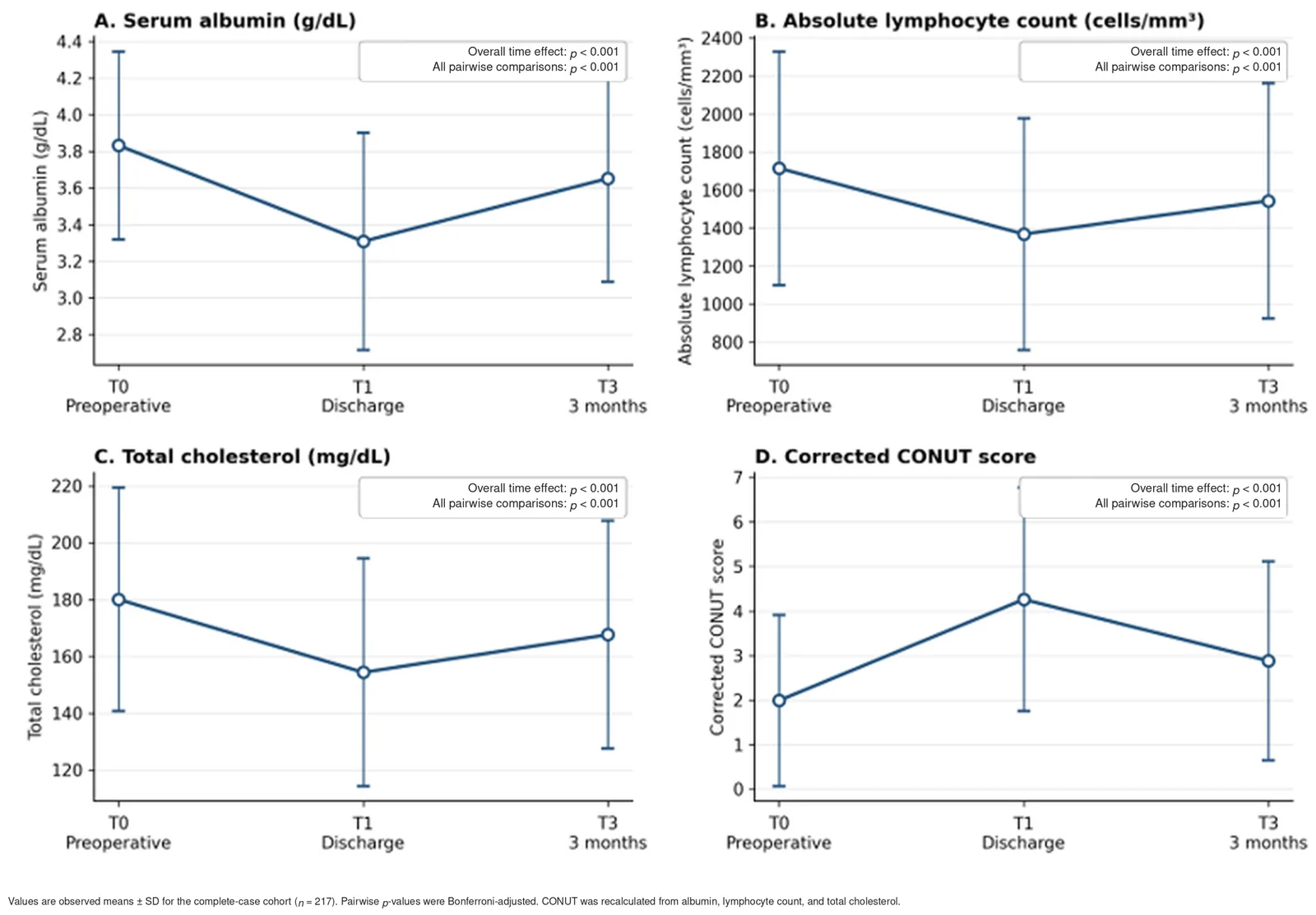
Longitudinal changes in nutritional–immune biomarkers following curative-intent gastrectomy for gastric cancer. Mean values (±standard deviation) are presented for (**A**) serum albumin, (**B**) absolute lymphocyte count, (**C**) total cholesterol, and (**D**) Controlling Nutritional Status (CONUT) score at the preoperative assessment (T0), hospital discharge (T1), and three-month postoperative follow-up (T3). All biomarkers demonstrated significant early postoperative changes in an adverse direction at hospital discharge, followed by partial recovery at three months. These early changes should be interpreted as reflecting the combined inflammatory, metabolic, immune, fluid-distribution, and nutritional response to surgery rather than acute malnutrition alone. Abbreviations: CONUT, Controlling Nutritional Status; T0, preoperative assessment; T1, hospital discharge; T3, three-month postoperative follow-up.

The overall time-effect *p*-values assess whether each biomarker differed across T0, T1, and T3, while the Bonferroni-adjusted pairwise *p*-values compare the respective assessment points. These *p*-values do not represent comparisons between subtotal and total gastrectomy [Table 2] [Figure 2].

To quantify the magnitude of recovery between discharge and the three-month assessment, the proportion of the initial postoperative decline regained by T3 was calculated descriptively. Recovery amounted to 65.6% for albumin, 50.5% for absolute lymphocyte count, and 51.7% for total cholesterol. After recalculation of CONUT from its constituent parameters, the corresponding recovery proportion was 60.9%. These findings indicate that biochemical nutritional recovery was already underway by three months but remained incomplete across all assessed domains.

### 3.3. Nutritional–Immune Biomarker Trajectories According to the Extent of Gastric Resection

After adjustment for age, sex, neoadjuvant chemotherapy, baseline body mass index, ASA III–IV status, pathological T3–T4 stage, and major postoperative morbidity, no significant time-by-gastrectomy interactions were identified for serum albumin. The adjusted difference in change between total and subtotal gastrectomy was −0.057 g/dL at hospital discharge (95% CI, −0.139 to 0.024; *p* = 0.166) and 0.056 g/dL at three months (95% CI, −0.025 to 0.137; *p* = 0.177). Similarly, total cholesterol trajectories did not differ significantly at hospital discharge (β = −1.11 mg/dL; 95% CI, −3.46 to 1.25; *p* = 0.356) or three months (β = 0.39 mg/dL; 95% CI, −1.96 to 2.75; *p* = 0.745).

A significant difference was observed in the early absolute lymphocyte response. Compared with subtotal gastrectomy, total gastrectomy was associated with an additional adjusted decline of 165.7 cells/mm^3^ at hospital discharge (β = −165.7; 95% CI, −204.9 to −126.6; *p* < 0.001). However, the time-by-gastrectomy interaction was no longer significant at three months (β = 1.1 cells/mm^3^; 95% CI, −38.0 to 40.3; *p* = 0.955), indicating convergence of lymphocyte counts during subsequent recovery.

After recalculation of CONUT from serum albumin, absolute lymphocyte count, and total cholesterol, no significant time-by-gastrectomy interactions were identified at hospital discharge (β = 0.11; 95% CI, −0.28 to 0.50; *p* = 0.576) or three months (β = −0.08; 95% CI, −0.47 to 0.31; *p* = 0.678). Sensitivity analyses replacing major postoperative morbidity with the occurrence of any postoperative complication yielded substantively consistent results. Therefore, total gastrectomy was associated with a transiently greater early lymphocyte decline but not with persistently less favorable albumin, cholesterol, or corrected CONUT trajectories.

**Figure 3:**
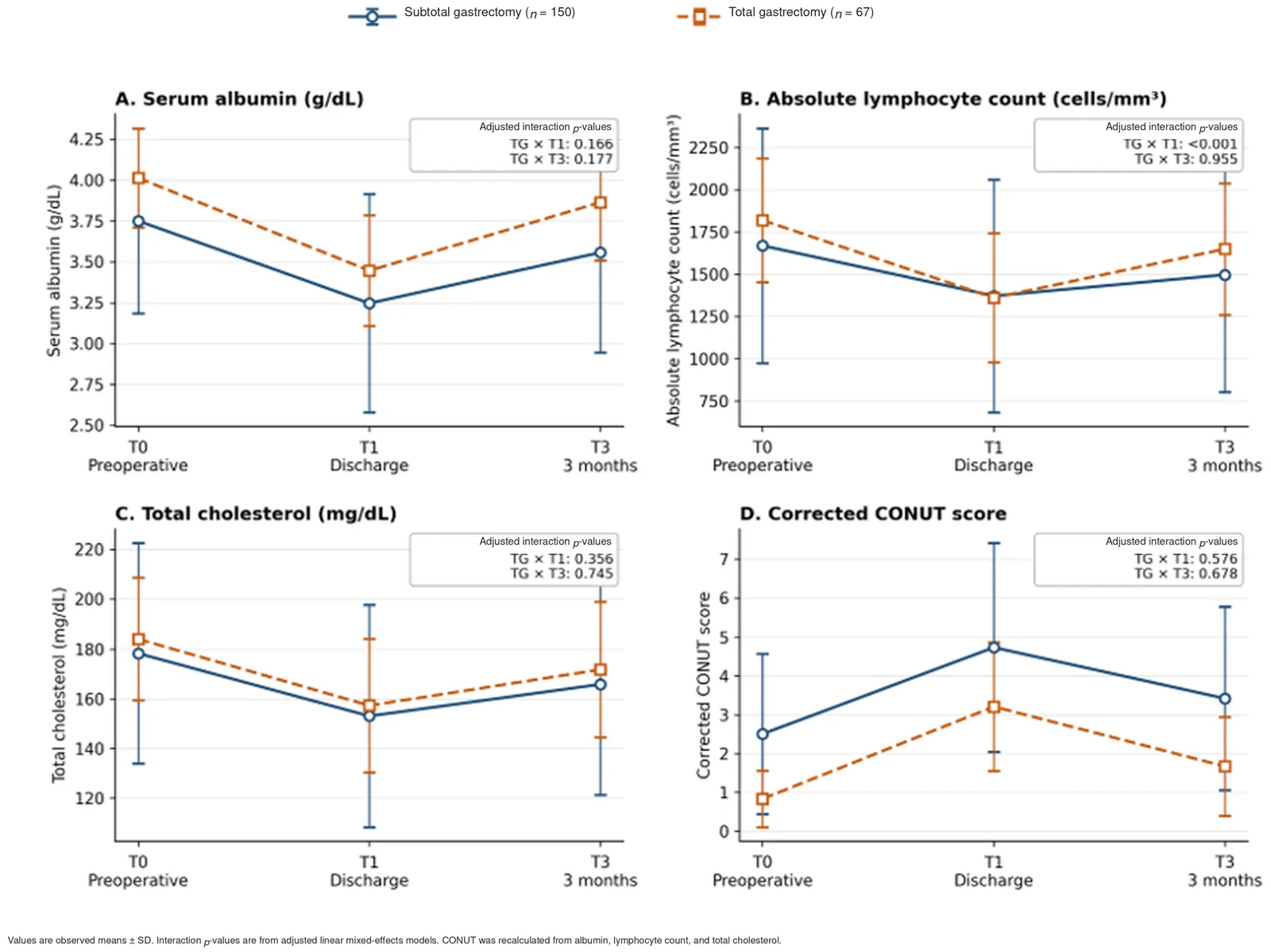
Observed longitudinal nutritional–immune biomarker trajectories according to gastrectomy type with adjusted interaction *p*-values. Observed mean trajectories with standard-deviation error bars are presented for (**A**) serum albumin, (**B**) absolute lymphocyte count, (**C**) total cholesterol, and (**D**) recalculated Controlling Nutritional Status (CONUT) score at the preoperative assessment (T0), hospital discharge (T1), and three-month follow-up (T3), stratified by subtotal and total gastrectomy. The plotted points and error bars represent observed means ± standard deviations. The displayed gastrectomy-type-by-time interaction *p*-values were obtained from linear mixed-effects models adjusted for age, sex, neoadjuvant chemotherapy, baseline body mass index, ASA III–IV status, pathological T3–T4 stage, and major postoperative morbidity. Time-by-stage and time-by-major-complication interactions were included. No significant procedure-related differences were identified for albumin, cholesterol, or corrected CONUT. Total gastrectomy was associated with a greater early lymphocyte decline at T1, but the between-procedure difference was no longer evident at T3.

Procedure-related differences were evaluated separately using gastrectomy-type-by-time interaction terms. These interaction coefficients represent the adjusted difference in change from T0 between total and subtotal gastrectomy at T1 and T3. Consequently, their *p*-values refer to between-group differences in biomarker trajectories rather than to within-group temporal changes [Table 3] [Figure 3].

## 4. Discussion

### 4.1. Principal Findings

This prospective longitudinal cohort study characterized early nutritional–immune biomarker changes after curative gastrectomy using repeated assessments and adjusted longitudinal modelling. A consistent pattern of marked adverse biomarker changes at hospital discharge followed by partial recovery at three months was observed. None of the evaluated parameters had returned completely to its preoperative level, indicating that postoperative metabolic, inflammatory, and nutritional adaptation continued beyond the immediate recovery period.

After adjustment for age, sex, neoadjuvant chemotherapy, baseline body mass index, ASA status, pathological tumor stage, and major postoperative morbidity, the extent of gastric resection was not associated with significantly different albumin, cholesterol, or corrected CONUT trajectories. Total gastrectomy was associated with a greater early decline in absolute lymphocyte count at hospital discharge; however, this difference was no longer evident at three months. These findings suggest a transient early immune–inflammatory perturbation rather than a generalized or sustained impairment of nutritional recovery attributable to total gastrectomy.

The contribution of the present study should be interpreted within its deliberately limited scope. The adverse nutritional and functional consequences of gastrectomy, including body-weight loss, sarcopenia, micronutrient deficiencies, and impaired quality of life, are already well established. The present analysis does not seek to reconfirm these long-term consequences. Its contribution lies in prospectively characterizing short-term trajectories of routine nutritional–immune biomarkers and examining whether the extent of gastric resection independently influences these trajectories after accounting for major differences in tumor stage and postoperative morbidity.

This adjusted analysis produced a more nuanced finding than the initial unadjusted comparison. Total gastrectomy was not independently associated with persistently less favorable albumin, cholesterol, or corrected CONUT trajectories. The only procedure-related difference was a greater early lymphocyte decline at hospital discharge, which was no longer evident at three months. These findings suggest that the apparent biochemical disadvantage associated with total gastrectomy may be explained partly by differences in disease severity and postoperative morbidity rather than by the anatomical extent of resection alone.

The transient early lymphocyte decline associated with total gastrectomy should not be interpreted as evidence of procedure-specific nutritional failure. Compared with subtotal gastrectomy, total gastrectomy generally involves a greater magnitude of tissue trauma and a more extensive lymphatic dissection, even within a D2 lymphadenectomy framework. These operative differences may produce a stronger perioperative inflammatory, neuroendocrine, and immunological response. Surgical stress may induce transient lymphopenia through lymphocyte redistribution, apoptosis, and inflammatory–neuroendocrine activation. Therefore, the greater decline observed at hospital discharge is more plausibly interpreted as a transient surgical–immunological response than as an isolated nutritional consequence of total gastric resection. The absence of a significant between-procedure difference at three months further supports this interpretation.

Collectively, these findings support the concept that postoperative biological and nutritional recovery is not a single postoperative event but a continuous biological process that depends on surgical stress, gastrointestinal adaptation, and nutritional support. The longitudinal study design offers a more complete picture of this recovery trajectory than cross-sectional postoperative assessments described in many prior studies [6,11,12,13,14,15,16,17,23].

### 4.2. Recovery Trajectories After Gastrectomy: Biological and Clinical Interpretation

The observed trajectories were characterized by marked adverse biomarker changes at hospital discharge followed by partial recovery at three months. Importantly, the early decrease in serum albumin should not be interpreted as a direct measure of acute nutritional depletion. Albumin is a negative acute-phase protein, and its concentration during the immediate postoperative period is strongly influenced by systemic inflammation, increased capillary permeability and redistribution into the extravascular compartment, perioperative fluid changes, and reprioritization of hepatic protein synthesis. Reduced oral intake, increased protein catabolism, and the metabolic demands of tissue repair may contribute simultaneously. Therefore, the T1 changes most appropriately represent the combined inflammatory, metabolic, and nutritional response to major surgery rather than isolated nutritional deterioration. These changes occur even in patients managed according to modern Enhanced Recovery After Surgery (ERAS) protocols and thus represent an expected component of postoperative adaptation rather than a failure of perioperative nutritional care [6,8,9,10,43].

Although there was obvious improvement in follow-up, recovery was incomplete at 3 months after surgery. This is in agreement with previous longitudinal studies showing that nutritional recovery after gastrectomy takes several months and depends on gradual intestinal adaptation and the resumption of oral intake and resolution of postoperative inflammation [14,15,16,17,23]. Oral nutritional supplementation has been shown to reduce postoperative weight loss and improve nutritional status in randomized controlled trials, but rarely completely restore preoperative nutritional reserves during the early recovery period [46,47,48]. Hence the present findings support current ESPEN recommendations for extended nutritional surveillance and individualized nutritional support following major upper gastrointestinal surgery rather than confining nutritional assessment to the immediate postoperative period [43]. Although perioperative recommendations were standardized, postoperative biological recovery may have been influenced by individual differences in dietary intake, tolerance of oral nutritional supplements, gastrointestinal symptoms, malabsorption, and adherence to dietetic recommendations. Therefore, the observed trajectories should not be attributed exclusively to anatomical or physiological consequences of the resection.

An important feature of the present study was the simultaneous longitudinal assessment of several routinely available nutritional–immune biomarkers. Nevertheless, none of these biomarkers is specific for malnutrition. Serum albumin is influenced predominantly by inflammation and illness severity during the acute postoperative period; lymphocyte count is affected by the immune and stress response; and cholesterol reflects several processes, including dietary intake, inflammation, hepatic metabolism, and metabolic adaptation. Because CONUT combines these variables, it inherits their sensitivity to systemic inflammation. These measures may therefore assist in describing postoperative biological recovery and identifying higher-risk patients, but they should be interpreted together with inflammatory markers, body-weight change, dietary intake, body composition, and clinical assessment rather than being used independently to diagnose malnutrition [11,12,13,30,34,35,36,37,38].

Statistical significance should be distinguished from clinical importance. Between T0 and T1, the mean absolute changes were −0.52 g/dL for albumin, −346 cells/mm^3^ for absolute lymphocyte count, −25.7 mg/dL for total cholesterol, and +2.27 points for CONUT. These changes were sufficiently large to cross commonly used CONUT component thresholds at the cohort level, indicating a substantial early postoperative biological perturbation. Nevertheless, such threshold crossing does not establish acute malnutrition because all three CONUT components are sensitive to inflammation and surgical stress.

For the adjusted comparison between procedures, total gastrectomy was associated with an additional early lymphocyte decline of 165.7 cells/mm^3^, corresponding to approximately 9.7% of the overall preoperative mean. However, no validated minimal clinically important difference has been established for postoperative lymphocyte trajectories following gastrectomy. Moreover, the between-procedure difference was absent at three months, and no significant procedure-related differences were identified for albumin, cholesterol, or corrected CONUT. The early lymphocyte finding should therefore be considered statistically robust but of uncertain independent clinical significance. Its clinical relevance requires confirmation through associations with patient-centered outcomes such as complications, treatment tolerance, physical function, quality of life, and long-term survival.

### 4.3. Clinical Implications

The findings of this study have several practical implications for the management of gastric cancer patients after surgery. First, the present findings indicate that biochemical recovery remained incomplete three months after surgery despite standardized perioperative management, emphasizing that hospital discharge should not be considered the end of nutritional rehabilitation. Instead, structured postoperative follow-up should combine clinical nutritional assessment, body-weight change, oral-intake evaluation, and validated nutritional screening tools. Routine biomarkers such as serum albumin and lymphocyte count may provide supplementary information but should be interpreted alongside inflammatory markers such as CRP, and should not be used independently to diagnose malnutrition [6,43].

Second, the adjusted analyses do not support the use of resection extent alone as a determinant of short-term nutritional surveillance intensity. Although total gastrectomy was associated with a greater early lymphocyte decline, this difference resolved by three months, while albumin, cholesterol, and corrected CONUT trajectories were not significantly different between procedures. Postoperative follow-up should therefore be individualized according to the combined clinical profile, including baseline nutritional status, tumor stage, postoperative morbidity, oral intake, body-weight loss, and the anticipated procedure-specific risk of micronutrient deficiency. Patients undergoing total gastrectomy still require systematic long-term surveillance for vitamin B12 and iron deficiency because of established physiological consequences of complete gastric resection [14,25,26,32,33].

The findings support continued clinical and biochemical assessment during early post-discharge follow-up. However, because follow-up was limited to three months and functional, body-composition, micronutrient, and patient-reported outcomes were not evaluated, the present results cannot define long-term gastric cancer survivorship needs. Comprehensive survivorship programs should integrate nutritional screening with body-weight monitoring, dietary assessment, body-composition evaluation, objective physical-function testing, micronutrient surveillance, and validated quality-of-life measures. Serial nutritional assessments may be incorporated into ERAS-based follow-up pathways to identify patients requiring additional nutritional, functional, or symptom-directed evaluation. Whether such risk-adapted multidisciplinary interventions improve long-term physical function or quality of life requires evaluation in prospective interventional studies [9,15,17,23,45,46,47,48].

Recovery after gastrectomy should be situated within a broader multidisciplinary rehabilitation framework. Gastrointestinal symptoms such as early satiety, dumping symptoms, reflux, nausea, altered bowel habits, abdominal pain, and constipation may impair oral intake, limit physical activity, and reduce quality of life. These effects may be compounded by comorbidities, chemotherapy, opioid analgesics, and antiemetic agents, all of which may contribute to gastrointestinal dysmotility and constipation in oncology patients. Accordingly, postoperative care may require coordinated input from surgeons, oncologists, clinical dietitians, rehabilitation physicians, physiotherapists, pain specialists, and psycho-oncology professionals.

Rehabilitation-based and integrative supportive-care strategies may have a role in selected symptomatic patients. A systematic review of oncology populations reported potential improvements in constipation-related symptoms and quality of life with physical exercise, abdominal massage, education regarding defecation posture, transcutaneous electrical nerve stimulation, acupuncture, and selected manual or osteopathic approaches. However, the evidence was heterogeneous, no standardized protocol was identified, and the included studies were not specific to patients following gastrectomy. These interventions should therefore be considered complementary and individualized rather than substitutes for established nutritional, pharmacological, oncological, or surgical management [49].

### 4.4. Strengths and Limitations

The strengths of this study include its prospective longitudinal design, standardized perioperative management, repeated assessment at predefined time points, and use of mixed-effects models accounting for within-patient correlation. The revised models also incorporated pathological tumor stage and postoperative morbidity, thereby addressing important differences in case mix between subtotal and total gastrectomy.

Nevertheless, the study has important limitations that substantially delimit its interpretation. First, the evaluated laboratory variables represent routinely available nutritional–immune biomarkers and not a comprehensive assessment of nutritional status. Body composition was not evaluated using computed tomography or bioelectrical impedance analysis; consequently, skeletal-muscle depletion and sarcopenia could not be examined. Objective functional outcomes, including handgrip strength, gait speed, physical-performance testing, or exercise capacity, were also unavailable. Dietary intake and gastrointestinal symptom burden were not systematically quantified, and patient-reported quality of life was not assessed using validated instruments. Furthermore, vitamin B12, iron indices, folate, vitamin D, and other relevant micronutrients were not measured longitudinally. Consequently, it was not possible to determine whether the observed biomarker changes correlated with preservation of skeletal-muscle mass, physical performance, treatment tolerance, functional independence, or health-related quality of life.

Second, follow-up was limited to three months. This period permits evaluation of early postoperative biochemical changes but is insufficient to characterize long-term nutritional adaptation, delayed micronutrient deficiencies, progressive sarcopenia, return to functional independence, or survivorship-related quality of life. The findings should therefore be described as early biomarker trajectories rather than long-term nutritional or functional recovery.

Third, the longitudinal biomarker analysis was restricted to 217 patients with complete assessments at all three time points. Among the 239 eligible patients, five died within 90 days and seventeen did not attend the three-month assessment. Because patient-level biomarker and clinical information was unavailable for these patients in the analytical dataset, informative attrition and survivorship bias cannot be excluded. Patients with fatal or otherwise unfavorable postoperative courses may consequently be underrepresented, potentially leading to an overestimation of nutritional recovery. The reported trajectories should therefore be interpreted as conditional recovery patterns among patients who survived and completed the three-month follow-up rather than as unconditional trajectories applicable to all patients undergoing gastrectomy.

Fourth is the absence of serial, concurrently measured inflammatory markers at all three assessment points. In particular, CRP could not be incorporated as a time-varying covariate in the longitudinal models. Therefore, the study cannot fully separate changes attributable to postoperative inflammation from those attributable to reduced intake or nutritional depletion. This limitation is especially important for the hospital-discharge measurements because albumin, lymphocyte count, and consequently CONUT are sensitive to the acute-phase and surgical-stress response. The T1 findings should therefore be interpreted as nutritional–inflammatory biomarker changes rather than as direct evidence of acute malnutrition.

Fifth, the discharge assessment was not obtained on an identical postoperative day for every participant. Because length of stay may be influenced by surgical complexity, complications, and the pace of clinical recovery, variation in T1 timing may have contributed to differences in biomarker values. Accordingly, T1 represents the biomarker status at discharge rather than a standardized postoperative nadir.

Within these limitations, the study provides a prospective description of early changes in routinely available nutritional–immune biomarkers during the first three months after gastrectomy. The findings should not be interpreted as a comprehensive assessment of nutritional or functional recovery. The principal adjusted result was that resection extent alone did not explain albumin, cholesterol, or corrected CONUT trajectories, whereas the procedure-related difference in lymphocyte response was transient. These findings generate the hypothesis that short-term postoperative biomarker changes are determined more strongly by the combined effects of tumor burden, postoperative morbidity, inflammation, and baseline clinical risk than by gastrectomy type alone.

Future prospective studies should extend follow-up to at least 12 months and combine routine laboratory biomarkers with serial CT- or BIA-derived body composition, muscle strength and physical-performance measurements, quantitative dietary assessment, vitamin B12 and iron indices, postoperative treatment exposure, and validated patient-reported outcome measures. Such multidimensional assessment is necessary to determine whether early biomarker trajectories predict clinically meaningful functional and survivorship outcomes.

## 5. Conclusions

This prospective longitudinal study characterized early nutritional–immune biomarker trajectories during the first three months following curative-intent gastrectomy. After adjustment for pathological tumor stage, baseline case mix, and postoperative morbidity, total gastrectomy was associated with a greater decline in absolute lymphocyte count at hospital discharge but not with persistently less favorable albumin, cholesterol, or corrected CONUT trajectories. The lymphocyte difference was no longer evident at three months.

These findings suggest that resection extent alone does not determine short-term biochemical recovery. The evaluated laboratory variables are inflammation-sensitive and do not provide a comprehensive assessment of nutritional status, body composition, physical function, micronutrient deficiency, quality of life, or long-term survivorship. Nevertheless, patients with persistent adverse biomarker changes, postoperative morbidity, reduced oral intake, or body-weight loss may warrant closer multidisciplinary nutritional and clinical surveillance. Prospective interventional studies incorporating nutritional, functional, body-composition, and patient-reported outcomes are required to determine whether risk-adapted nutritional and rehabilitation strategies improve postoperative recovery.

## Figures and Tables

**Table 1:** Characteristics of the complete-case longitudinal cohort according to the extent of gastric resection (*n* = 217).

Characteristic	Overall (*n* = 217)	Subtotal Gastrectomy (*n* = 150)	Total Gastrectomy (*n* = 67)	*p*-Value
Age (years)	61.4 ± 10.6	61.5 ± 10.8	61.2 ± 10.2	0.844
Male sex, *n* (%)	128 (59.0)	91 (60.7)	37 (55.2)	0.451
Body mass index (kg/m^2^)	23.8 ± 3.5	23.7 ± 3.5	23.9 ± 3.5	0.606
ASA class III–IV, *n* (%)	77 (35.5)	59 (39.3)	18 (26.9)	0.076
Neoadjuvant chemotherapy, *n* (%)	83 (38.2)	47 (31.3)	36 (53.7)	0.002
Pathological T3–T4 stage, *n* (%)	123 (56.7)	73 (48.7)	50 (74.6)	<0.001
Node-positive disease (pN1–3), *n* (%)	161 (74.2)	112 (74.7)	49 (73.1)	0.812
D2 lymphadenectomy, *n* (%)	151 (69.6)	84 (56.0)	67 (100.0)	<0.001
R0 resection, *n* (%)	203 (93.5)	136 (90.7)	67 (100.0)	0.006
Operative time (min)	214.6 ± 35.9	201.2 ± 23.8	244.7 ± 40.3	<0.001
Estimated blood loss (mL)	313.9 ± 95.7	325.1 ± 81.6	288.9 ± 118.5	0.025
Any postoperative complication, *n* (%)	128 (59.0)	72 (48.0)	56 (83.6)	<0.001
Major postoperative complication, Clavien–Dindo ≥ II	48 (22.1)	30 (20.0)	18 (26.9%)	0.343
Length of hospital stay (days)	11 (9–14)	12 (9–14)	10 (8–13)	0.024
Postoperative weight loss at 3 months (%)	10.5 ± 5.7	10.3 ± 6.2	11.1 ± 4.3	0.281

Values are presented as mean ± standard deviation (SD), median (interquartile range [IQR]), or number (percentage), as appropriate. Continuous variables were compared using the independent-samples *t*-test or Mann–Whitney *U* test according to data distribution, whereas categorical variables were compared using the χ^2^ test or Fisher’s exact test. ASA, American Society of Anesthesiologists. Because the surgical procedure was selected according to oncological and anatomical criteria, the subtotal and total gastrectomy groups were not assumed to be exchangeable at baseline. Between-group differences were examined descriptively and statistically and were subsequently considered in the adjusted longitudinal models.

**Table 2:** Longitudinal evolution of nutritional–immune biomarkers following gastrectomy.

Variable	Preoperative (T0)	Hospital Discharge (T1)	3 Months (T3)	Overall *p*-Value †	T0 vs. T1	T1 vs. T3	T0 vs. T3
Serum albumin (g/dL)	3.83 ± 0.51	3.31 ± 0.59	3.65 ± 0.56	<0.001	<0.001	<0.001	<0.001
Absolute lymphocyte count (cells/mm^3^)	1715 ± 614	1369 ± 609	1544 ± 619	<0.001	<0.001	<0.001	<0.001
Total cholesterol (mg/dL)	180.1 ± 39.3	154.4 ± 40.1	167.7 ± 40.0	<0.001	<0.001	<0.001	<0.001
CONUT score	1.99 ± 1.92	4.26 ± 2.51	2.88 ± 2.23	<0.001	<0.001	<0.001	<0.001

The overall *p*-value evaluates the effect of time in the complete-case cohort. Pairwise *p*-values represent Bonferroni-adjusted comparisons between assessment points and should not be interpreted as comparisons between gastrectomy groups. † Overall *p*-values were obtained from linear mixed-effects models with patient-specific random intercepts. Pairwise comparisons were adjusted using the Bonferroni correction. Values are presented as mean ± standard deviation (SD). Abbreviations: CONUT, Controlling Nutritional Status score.

**Table 3:** Adjusted time-by-gastrectomy interactions for longitudinal nutritional–immune biomarker trajectories.

Outcome	Interaction Term	Adjusted β	95% CI	*p*-Value
Serum albumin (g/dL)	Total gastrectomy × T1	−0.057	−0.139 to 0.024	0.166
	Total gastrectomy × T3	0.056	−0.025 to 0.137	0.177
	pT3–T4 stage × T1	−0.017	−0.093 to 0.058	0.650
	pT3–T4 stage × T3	−0.043	−0.119 to 0.032	0.262
	Major complication × T1	−0.021	−0.109 to 0.066	0.633
	Major complication × T3	−0.010	−0.098 to 0.078	0.829
Absolute lymphocyte count (cells/mm^3^)	Total gastrectomy × T1	−165.7	−204.9 to −126.6	<0.001
	Total gastrectomy × T3	1.1	−38.0 to 40.3	0.955
	pT3–T4 stage × T1	16.5	−19.9 to 53.0	0.374
	pT3–T4 stage × T3	6.6	−29.9 to 43.0	0.724
	Major complication × T1	−3.8	−46.1 to 38.5	0.861
	Major complication × T3	−9.3	−51.6 to 33.0	0.668
Total cholesterol (mg/dL)	Total gastrectomy × T1	−1.11	−3.46 to 1.25	0.356
	Total gastrectomy × T3	0.39	−1.96 to 2.75	0.745
	pT3–T4 stage × T1	−1.74	−3.93 to 0.45	0.119
	pT3–T4 stage × T3	−1.12	−3.31 to 1.07	0.317
	Major complication × T1	0.00	−2.54 to 2.55	0.997
	Major complication × T3	0.28	−2.26 to 2.82	0.830
Corrected CONUT score	Total gastrectomy × T1	0.11	−0.28 to 0.50	0.576
	Total gastrectomy × T3	−0.08	−0.47 to 0.31	0.678
	pT3–T4 stage × T1	0.07	−0.30 to 0.43	0.721
	pT3–T4 stage × T3	−0.04	−0.40 to 0.32	0.829
	Major complication × T1	0.26	−0.16 to 0.68	0.229
	Major complication × T3	0.23	−0.19 to 0.65	0.289

Values represent fixed-effect estimates from linear mixed-effects models with patient-specific random intercepts. Time was treated as a categorical variable, with T0 as the reference category. Models included gastrectomy type, age, sex, neoadjuvant chemotherapy, baseline body mass index, ASA III–IV status, pathological T3–T4 stage, and major postoperative morbidity, defined as Clavien–Dindo grade III or higher. Time-by-gastrectomy, time-by-stage, and time-by-major-complication interactions were included. The coefficients for total gastrectomy × T1 and total gastrectomy × T3 represent adjusted differences in change from T0 between total and subtotal gastrectomy. The corresponding *p*-values test whether the longitudinal change differs significantly between procedures.

## Data Availability

The datasets generated and/or analyzed during the current study are available from the corresponding author upon reasonable request, subject to institutional and ethical restrictions protecting patient confidentiality.

## Abbreviations

The following abbreviations are used in this manuscript:

ASA	American Society of Anesthesiologists
BMI	Body Mass Index
CI	Confidence Interval
CONUT	Controlling Nutritional Status
ERAS	Enhanced Recovery After Surgery
ESPEN	European Society for Clinical Nutrition and Metabolism
GLIM	Global Leadership Initiative on Malnutrition
GNRI	Geriatric Nutritional Risk Index
IQR	Interquartile Range
NCCN	National Comprehensive Cancer Network
PNI	Prognostic Nutritional Index
SD	Standard Deviation
STROBE	Strengthening the Reporting of Observational Studies in Epidemiology
T0	Preoperative assessment
T1	Hospital discharge assessment
T3	Three-month postoperative follow-up
